# Evaluation of Spatio-Temporal Patterns of Predation Risk to Forest Grouse Nests in the Central European Mountain Regions

**DOI:** 10.3390/ani11020316

**Published:** 2021-01-27

**Authors:** Jan Cukor, Rostislav Linda, Oddgeir Andersen, Lasse Frost Eriksen, Zdeněk Vacek, Jan Riegert, Martin Šálek

**Affiliations:** 1 Forestry and Game Management Research Institute, v.v.i., Strnady 136, 252 02 Jíloviště, Czech Republic; lindar@fld.czu.cz; 2 Faculty of Forestry and Wood Sciences, Czech University of Life Sciences Prague, Kamýcká 129, 165 00 Prague 6, Czech Republic; vacekz@fld.czu.cz; 3 Department of Terrestrial Ecology, Norwegian Institute for Nature Research, 7034 Trondheim, Norway; oddgeir.andersen@nina.no (O.A.); lasse.eriksen@nina.no (L.F.E.); 4 Department of Zoology, Faculty of Science, University of South Bohemia, Branišovská 1760, 370 05 České Budějovice, Czech Republic; honza@riegert.cz; 5 Institute of Vertebrate Biology, The Czech Academy of Sciences, Květná 8, 603 65 Brno, Czech Republic; martin.sali@post.cz; 6 Faculty of Environmental Sciences, Czech University of Life Sciences Prague, Kamýcká 1176, Suchdol, 165 21 Prague, Czech Republic

**Keywords:** artificial nests, nest predation, camera-trapping, forest grouse conservation, wildlife management

## Abstract

**Simple Summary:**

Forest grouses are among the most endangered ground-nesting birds in Central Europe. Their rapid population decline was associated with habitat loss and increasing predation risk leading to low breeding success. The aim of this study was to describe black grouse nest predators and potential predation risk in a study area with a small, extant population of black grouse (Ore Mts.) and in a study area with an already extinct grouse population (Jeseníky Mts.) in the Czech Republic. In order to determine the predation intensity to black grouse nests, 50 artificial nests (28 in Ore Mts., 22 in Jeseníky Mts.) were monitored using camera traps. The results showed that 56% of nests were predated. Within the time needed for successful incubation of the eggs (25 days), the nest survival probability was on average 45.5%. The proportion of depredated nests did not differ between habitat types (i.e., open forest interior, clearing, forest edge). The stone marten was the main potential nest predator in both study areas (39% in total), followed by common raven (25%) and red fox (22%). In conclusion, our study revealed the high predation pressure on black grouse nests which corresponds with increasing population trends of mesopredators and wild boars in Central Europe.

**Abstract:**

We evaluated the spatiotemporal patterns of predation risk on black grouse nests using artificial nests that were monitored by camera traps in mountain areas with a small extant (Ore Mts.) and already extinct (Jeseníky Mts.) black grouse population. The overall predation rate of artificial nests was 56% and we found significant differences in survival rate courses over time between both study areas (68% Ore Mts. vs. 41%, Jeseníky Mts.). Within the time required for successful egg incubation (25 days), nest survival probability was 0.32 in the Ore Mts. and 0.59 in Jeseníky Mts. The stone marten (*Martes foina*) was the primary nest predator in both study areas (39% in total), followed by common raven (*Corvus corax*, 25%) and red fox (*Vulpes vulpes*, 22%). The proportion of depredated nests did not differ between habitat types (i.e., open forest interior, clearing, forest edge), but we recorded the effect of interaction of study area and habitat. In Ore Mts., the main nest predator was common raven with seven records (37%). The Eurasian jay (*Garrulus glandarius*) was responsible for most predation attempts in Jeseníky Mts. (five records, i.e., 83%), while in the Ore Mts., most predation attempts were done by red fox (six records, i.e., 38%).

## 1. Introduction

Ground-nesting bird species populations have dramatically decreased during recent decades in Europe [1,2,3] with most dramatic declines reported for larger species such as waders and bustards inhabiting agricultural landscape [2,4,5,6] or forest grouses (*Tetraonidae*) [7,8,9,10,11,12]. For example, the long-term population decline and range contraction of black grouse (*Lyrurus tetrix*) has been recorded in the majority of its distribution area, including the British Isles, continental Europe, and Fennoscandia [7,8,12,13,14,15,16]. The reasons for population declines are multifactorial, however, they are mainly related to land-use intensification and habitat loss [8,14], climatic change [8], human activities [14,17], and increased predation risk [8,16,18,19]. In small and isolated populations low genetic variability may also play a role in population declines [20,21]. The predation of adult birds, nests, and chicks is among the most important reasons of black grouse population decline [8,16,18]. The reasons for adult bird mortality are relatively well-researched, as predation has been found to be the proximate cause of mortality in adult black grouse [8,19]. Although predation is the main source of mortality of black grouse, the predation rate may significantly differ across the grouse distribution range and locally may be influenced by site-specific habitat characteristics, black grouse population density, and predator composition [8]. For example, mammalian predators were a major cause of adult black grouse mortality in the Czech Republic [22], whereas avian predators were identified as most important cause of adult grouse mortality in Finland [19]. In particular, more than half of radio-tagged black grouse females were depredated by avian predators in the study by Pekkola et al. [19]. However, the adult survival seems to be stable and, in some populations, even increasing [8].

Nest success and chick survival seem to be crucial for the reduction of black grouse population loss. Jahren et al. [8] found that nest success declined from 90% to 55% between 1934 and nowadays in Fennoscandia. The chick survival rate decreased as well, for example, the black grouse hens had on average 3.3 chicks fledged in 1971 decreasing to on average 1.2 chicks in 1988 in Central Europe. This trend is comparable with the situation in the British Isles, where the number of chicks per black grouse hen decreased from 2.0 chicks in 1992 to 1.3 in 2001 on average [8]. The nest predation is often considered to be the most significant factor responsible for low nest success of ground-nesting birds, and of black grouse [23,24], and the predator composition and population density is closely related to chick survival rates.

Currently, the black grouse distribution is mainly restricted to mountain regions in Central Europe [20,21,25,26]. The occurrence of black grouse is mainly linked with the presence of open mixed or coniferous woodland environments, peat bogs, wet meadows, clearings, and other early successional habitats [25,27]. Due to the rapid population decline and lower numbers of black grouse individuals [28], it is complicated to evaluate the predation risk to real nests in fragmented subpopulations in Central Europe. Therefore, artificial nests were used as was done in previous studies [24,26,29,30]. Artificial nests are commonly used to examine the effect of nest predation on populations of studied species as finding real nests of ground-nesting birds, and forest grouses in particular, is challenging [31,32,33]. However, the absolute rate of nest predation on artificial nests may differ from predation on natural nests. Hence, the results and management implications based on experiments with artificial nests should be evaluated with caution [31,32,34,35]. Still, artificial nest experiments may provide important data about predator composition in the study localities and relative rates of nest predation over space and time [29,36].

During recent decades, many studies were performed on predation rate on ground-nesting forest grouses using artificial nests [24,26,37]. The nest predators were mostly identified by tooth or beak marks left on the wax eggs, however, it was not possible to identify avian predator species and the time of predation this way [24,30,38]. Therefore, the aims of this study were (i) to identify main predators and assess predation rate on artificial nests mimicking black grouse nests by camera-trapping, (ii) to compare the predation rate in two mountain areas with residual and already extinct black grouse populations, (iii) to compare predation rate in different habitat types (open forest, clearing, and forest edge) and predation times during the day, and finally (iv) to assess differences in behavior among predator species.

## 2. Materials and Methods

### 2.1. Study Area

The study was conducted in two different study areas in mountain regions of the Czech Republic, Central Europe. The first study area was situated in the east part of Ore Mts. in the wider area of Cínovec moorland (N 50°42.74′, E 13°44.35′). The acreage of the area of interest where the artificial nests were placed was about 400 ha with altitudes ranging between 850 and 890 m a.s.l. The abundance of black grouse males fluctuated between 14 and 24 individuals in previous years (2016–2019) in the whole Cínovec area in Ore Mts. [39]. Due to low population density of the black grouse in the study area, the prevailing mating strategy is solitary display and therefore individual males are not clustered in the leks, which is described e.g., by Höglund and Stöh [40]. The occurrence of other forest grouses (i.e., hazel grouse *Bonasia bonasia* and capercaillie *Tetrao urogallus*) in the study area was not recorded during the last four decades [28]. Forest stands consist of Norway spruce (*Picea abies*) [41] and non-native silver spruce (*Picea pungens*) which was planted in Ore Mts. as a stress tolerant tree species into the pollution-damaged ecosystem [42,43]. The broadleaf tree species are mainly represented by downy birch (*Betula pubescens*) and common rowan (*Sorbus aucuparia*) [44]. The supplementary feeding of wild boars (*Sus scrofa*) is a common hunting management practice in the study area throughout the whole year with on average one feeding site per 100 ha. The average number of hunted wild boars is 2.3 ind. per 100 ha [45].

The second study area was situated in Jeseníky Mts. close to Králický Sněžník (N 50°10.97′, E 16°52.31′). The area of the Jeseníky Mts. where the artificial nests were placed was about 700 ha and the average altitudes ranged from 680 to 1230 m a.s.l. The black grouse population was present here in the second part of the 20th century, and the population became extinct between 1970 and 1980 [46]. The population of capercaillie rapidly declined and total population size was estimated to 16 individuals in the whole Jeseníky Mts. (ca. 530 km^2^) in 1999 with no occurrence in the study area. A small population of hazel grouse is distributed across the Jeseníky Mts., however due to discrete behavior, the population numbers and density in the study area are unknown [47]. The forests are represented by open mountain forest with Norway spruce and interspersed broadleaf species like common rowan, silver birch (*Betula pendula*), or sycamore maple (*Acer pseudoplatanus*). Supplementary feeding of wild boars was banned due to possible attraction of nontargeted species like red fox (*Vulpes vulpes*) or common raven (*Corvus corax*) [48,49] into the area where the black grouse reintroduction project has been in process since 2019 (project n. TH04030524: Model of conservation and development of habitat and population of Tetraonidae in the Králický Sněžník area). The mean number of hunted wild boars is 0.6 ind. per 100 ha [45].

### 2.2. Experimental Design

We used artificial nests for the evaluation of predation rate on black grouse nests. Artificial ground nests were installed for 30 consecutive days. Three brown small-sized domestic hen eggs were used in each artificial nest, similarly to previous studies [24,26,29,30]. To minimize human scent, we handled the nests and eggs with latex gloves [50]. Eggs were placed onto a simple hand-made depression on the ground, without any added material [30]. Artificial nests were placed into three habitat types: (i) open forest interior (i.e., at least 50 m inwards from the main edge of forest with low tree density), (ii) forest edge (i.e., up to 20 m from the forest edge), and (iii) clearing (i.e., at least 50 m from the forest edge into the clearing). The accurate nest location in a particular habitat type was selected randomly, imitating the distribution of natural nests which are also commonly distributed quasi randomly [51]. The artificial nests were installed between 26 April and 14 May in 2020, which corresponds to the peak of breeding season of black grouse [52]. In total, 28 artificial nests (10 in open forest interior, 9 in clearing, and 9 in forest edge) were installed in the Ore Mts. (with black grouse population) and 22 artificial nests (6 in open forest interior, 6 in clearing, and 10 in forest edge) were installed in Jeseníky Mts. (without black grouse population). The nests were considered depredated when at least one of the three placed eggs was destroyed or missing [53,54] (see below).

### 2.3. Potential Predator Monitoring

The monitoring of nest predation rates and potential predator behavior was realized using camera traps UO Vision UV 595 HD (UOVision Technology CO. LTD., Shenzhen, China) with an invisible IR camera (12 megapixels), trigger speed of 0.65 s, and HD video (1080p) recording (for more information see www.uovision.com). All cameras (one camera per each nest) were installed on a tree or stone at the distance of 4–8 m from the artificial nest. The date and time were recorded automatically at the beginning of each video. The sites were checked every 7–14 days to download recorded videos from the camera traps. The content of artificial nests was checked as well. The game camera started the video automatically when motion was detected and were set to record 30-s videos. The following types of potential predator behavior were recorded according to Bu et al. [55]: (i) predation (i.e., predation of artificial eggs), (ii) predation attempts (i.e., pecking into an egg, consumption of the leftovers after primary predation, noising around the eggs etc.), (iii) pass-by events (i.e., ignoring the eggs, in most cases probably due to the long distance from the nest). The detected predation occurrences were further sorted to (i) primary predation (the first predation occurrence captured on camera for each particular nest) and (ii) repeated predation. Repeated predation was not considered in survival analysis (as each nest could be only depredated once) and served only for analysis of predator sequence and time of potential predator occurrence. The videos were manually inspected, and the records were summarized in table for further statistical analyses. The numbers of predated and nonpredated nests according to video analysis are mentioned in Table 1.

### 2.4. Statistical Analyses

The proportion of depredated nests in both study areas was calculated. Confidence intervals (CI) were computed in R software 4.0.2 [56] by survival probability function using the “survival” package [57].

The comparison of the course of cumulative nests’ survival during the time was done using Cox proportional hazards analysis—type 3 tests [58]. Data unit represented each installed nest (n = 50). Since not all nests were predated, we used right-censoring for these cases. For predated nests, we used only the first predation event. For first predation event on each of the predated nests, we calculated the number of days from nest installation. The following independent variables were tested: habitat type (forest edge, forest interior, and clearing), study area (Jeseníky and Ore Mts.), and their interaction. The following partial post-hoc tests of the effect of study area within each habitat category were computed using log-rank tests [56]. In the graph on differences between the courses of cumulative survival during the time between the study areas, we indicated the time needed for incubation 25 days [59] and extracted actual survival rate for each study area. To construct the survival curves, we used a Kaplan–Meier method [59]. All these analyses were performed using Statistica 13 software [60].

To test the differences in behavior of predator species by the nests within study areas and across habitats, we used multivariate canonical correspondence analysis (CCA) using Canoco 5 software [61]. Data unit was represented by events represented by predation, predation attempts and pass-by events (*n* = 82). We used presence/absence of each predator (Eurasian jay *Garrulus glandarius*, common raven, wild boar *Sus scrofra*, red fox, Eurasian pine marten *Martes martes*, stone marten *Martes foina*, and European badger *Meles meles*) as response variables. Independent variables were represented by study area (Jeseníky and Ore Mts.), habitat (forest edge, forest interior, and clearing), and category of behavior of predator by nest (predation events, predation attempts, and pass-by events).

The sequence of predators at nests was also computed using CCA analysis, where data unit represented predation events represented by predation and repeated predation (*n* = 50). Response variables were presence/absence of each predator species by the nest. Study area and habitat type were used as covariates, we tested the effect of sequence of predators. The statistical significance in both CCA analyses was obtained by Monte-Carlo permutation tests under 499 permutations.

For analysis of potential predator record time during the day, the records were grouped into two groups—avian (*n* = 28) and mammalian species (*n* = 79). The difference in their occurrence in day and night hours (according to accurate sunrise and sunset in particular days; Central European Time) was tested using generalized linear mixed models (GLMMs) in R 4.0.2 software. All types of records (predation, repeated predation, predation attempt, or pass-by events) were included in the analysis (*n* = 107). We used lmer function with logit link function for binomial distribution of dependent variable (bird/mammalian predator). Study area and habitat type were used as variables with random effect. We tested the difference between day (5:00 a.m.–21:59 p.m.) and night (22:00 p.m.–4:59 a.m.) events. We also tested the effect of real time of recording. Due to collinearity, we tested the effects of these variables separately. Firstly, we built a null model without factors and then we tested the effect of each independent variable against the null model using anova function. Since real time data were on a circular scale, we used transformed values [62]. For each time, we calculated two values (x—hour and y—hour) that defined the time based on angle on circular scale. Calculations were done after the following equations: x—hour = sin (2π*(time/24)), y—hour = cos (2π*(time/24)). Interaction of—hour and y—hour was used as independent time variable. In the graph, we show nontransformed hours.

## 3. Results

**Figure 1:**
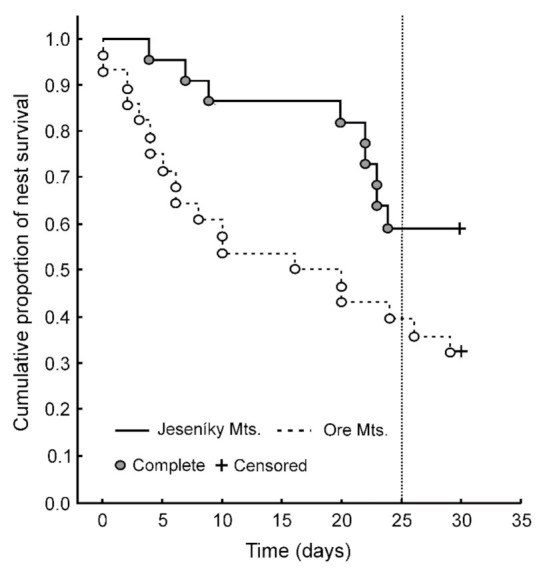
Cumulative proportion of nest survival in Jeseníky and Ore Mts. during the study period. The dashed vertical line at 25 days (incubation time) shows different survival rate for Jeseníky Mts. (0.59) and Ore Mts. (0.32). Kaplan–Meier method was used to fit the curves. Complete (predated) and censored (non-predated) nests are indicated.

In total, we recorded 28 of 50 artificial nests depredated in both study areas during a 30-day period which corresponds to relatively high predation rate (56%). In particular, 19 of 28 (68%; 95% CI: 48–84%) artificial nests were depredated in the Ore Mts. and 9 out of 22 (41%; 95% CI: 21–64%) artificial nests were depredated in the Jeseníky Mts. The comparison of nest survival course between study areas showed a significant difference (Table 2). The survival probability showed a relatively stable decrease in the case of the population in Ore Mts. In contrast, a steep drop in survival probability was observed from around day 22 from egg setting in Jeseníky Mts. (Figure 1). After the time required for black grouse egg incubation (25 days; [63]), the survival probability was higher in Jeseníky Mts. (0.59) compared to Ore Mts. (0.32, Figure 1).

**Figure 2:**
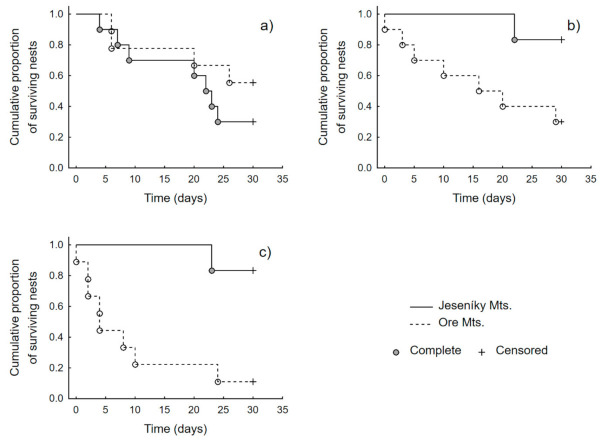
Cumulative proportion of surviving nests within forest edge (**a**), forest interior (**b**), and forest clearings (**c**) separately for Jeseníky and Ore Mts. Kaplan–Meier method was used to fit the survival curves, complete (predated) and censored (non-predated) nests are indicated.

We did not find significant differences in survival rate courses during the time among habitat types, but we found a significant effect of interaction between study area and habitat type (Cox proportional hazards analysis, Table 2). Further analyses showed that the statistical difference was not found between the courses of cumulative survival of nests in time for forest edges of Jeseníky and Ore Mts. (Log-rank test, Test statistic = 1.05, *p* = 0.296, Figure 2a). However, we found significant differences between study areas in the courses of cumulative survival of nests in time for forest clearings (Log-rank test, Test statistic = −2.81, *p* = 0.005) and forest interior (Log-rank test, Test statistic = −2.12, *p* = 0.034). However, it is important to note that in both above-mentioned significant analyses in Jeseníky Mts. there was only one predated nest compared to more predated nests in Ore Mts. In both cases, these single nests were predated after 20 days of their installation compared to earlier predation events in Ore Mts. (Figure 2b,c).

**Figure 3:**
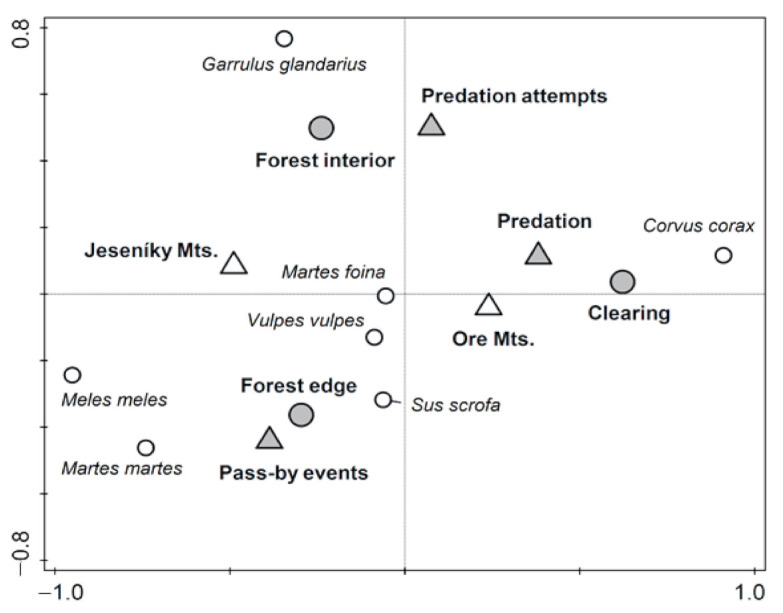
Projection scores for each recorded predator species by artificial nests in Jeseníky and Ore Mts. with respect to three habitats and predators’ behavior. CCA analysis, I. and II. ordination axes together explain 65.3% of variability.

Based on results of multivariate analysis, the main differences in distribution of recorded predators were found among habitats. The predators’ behavior was also significantly linked with each predator species (Table 3). The analysis also uncovered relationships among these tested variables. Predation events were more often recorded on clearings in Ore Mts. and attempts to predate the nest were more obviously recorded in the forest interior of Jeseníky Mts. Mammalian predators were concentrated along forest edges, while the predation by common raven was recorded in the forest clearings. Finally, pass-by events were more common in forest edges and predation attempts by Eurasian jay in the forest interiors (Figure 3).

**Figure 4:**
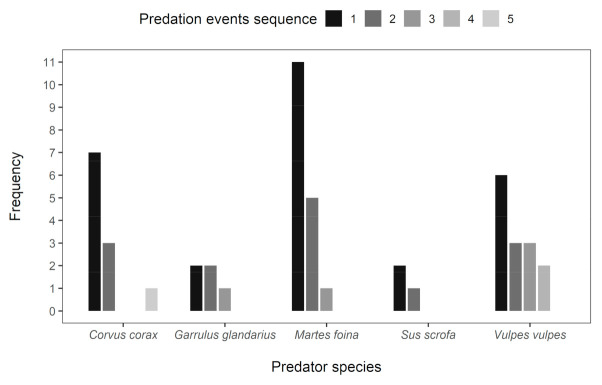
Predation events sequence recorded at artificial nests in both study areas together. Only predator records during predation events are presented (not predation attempts or individuals passing by).

The relationships among tested variables had a direct link to distribution of recorded predator species. The stone marten was the main nest predator in Jeseníky (89% of predation events), the red fox was recorded in one case of nest predation (11%). In Ore Mts., the main nest predator was common raven with seven records (37%). The Eurasian jay was responsible for most predation attempts in Jeseníky (five records, i.e., 83%), while in the Ore Mts., most predation attempts were done by red fox (six records, i.e., 38%). Red fox was also the most often detected animal when counting recorded pass-by events in both study areas (seven in Jeseníky, i.e., 58% and eight in Ore Mts., i.e., 40%, Figure 4, Table 4). All predators were detected individually, except for wild boars, which visited artificial nests also in family groups.

From the analysis of predator occurrence at the nests, we also assessed the sequence of detected predators at each particular nest. Multivariate analysis of the sequence of visiting the artificial nests did not show significant differences among predator species (CCA analysis, 40.2% of explained variability, pseudo-F = 1.6, *p* = 0.152). Despite this nonsignificant result, we found in the dataset the following patterns. The mammalian predators were the most common predators observed later on at the nests (Figure 4). Stone marten was observed as the first predator at the nest in most cases followed by common raven and red fox. Stone marten was also recorded as a second predator at the nest in most cases. Common raven was as a second predator at the nest recorded with the same frequency as the red fox, but was not recorded as the third or fourth predator, contrary to red fox, which was observed much more frequently during repeated predation. Eurasian jay and wild boar were both observed occasionally during repeated predation (Figure 4). On average, each nest was visited by a predator (predation or scavenging) 1.8 times (95% CI: 1.4–2.2).

**Figure 5:**
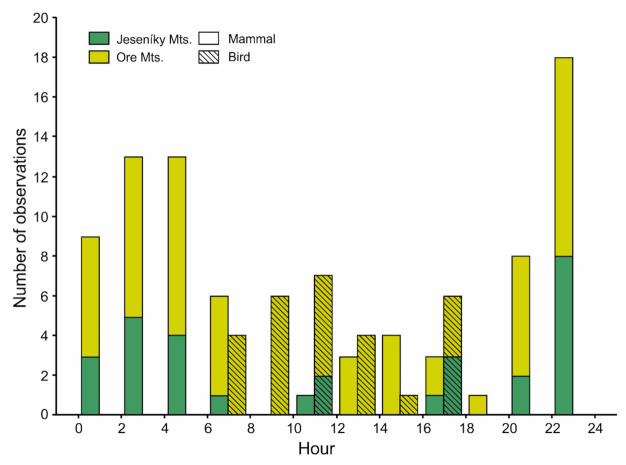
Distribution of recorded bird and mammalian predators at nests during the day (*n* = 107 events).

Mammals were mostly recorded during night hours, whereas records of avian predators were restricted to the daytime (Figure 5). We revealed a statistically significant relationship between predator type and time of record (day/night, Table 5). We observed 7 out of 25 records (28%) of mammals during the daytime in Jeseníky Mts. and 23 out of 54 records (43%) in Ore Mts. Independently, we found significant differences between presence of bird and mammalian predators at nests during the course of the day (Table 5, Figure 5).

## 4. Discussion

The average predation rate of artificial nests in our study reached 56% in both study areas combined. There was a significant difference between the rate of depredated nests in the study area with the residual black grouse population (68%, Ore Mts.) and the study area with a locally extinct black grouse population (41%, Jeseníky Mts.). In general, the predation of artificial nests by mammalian predators was more frequent (19 out of 28 cases, 67.9%) than the predation by avian predators (32.1%). The overall number of detected mammalian predators (61 cases) was also higher compared to avian predators (21 cases); however, this result may be caused by general higher detectability of mammalian predators with ground-dwelling activity. Regarding the analysis of predator activity during the day, the mammalian predators were mostly detected at night contrary to avian predators.

The overall observed predation rate in both study areas was much higher in comparison with previously conducted research in the Czech Republic [24], which was done 12 km west of our study area in Ore Mts. In this study, the overall predation rate on artificial nests was relatively low (i.e., 17.7%). On the other hand, another study conducted in south-western Poland (on the border with Czech Republic; [26]) found that all of 100 artificial nests placed in the area with a black grouse population were depredated within 7 days. The major differences between our findings and the two previous studies with contrasting results [24,26], suggests that there may be large differences in the site-specific predator composition and population density (e.g., due to landscape structure or game management).

Our result showed that there was a constant decrease in nest survival probability in the Ore Mts., while in the Jeseníky Mts. area, the survival rate was quite high until a relatively steep drop was observed after day 20. This could be caused by lower numbers of predators in this study area and, thus, longer time required for detection of the nests. Nevertheless, after 25 days (i.e., time needed for successful incubation of black grouse eggs), the survival probability was 0.59 in Jeseníky Mts. and 0.32 in Ore Mts. These numbers support previous findings about the role of predators in nesting success of forest grouse [8].

The predation on artificial nests was similar across all habitat types (i.e., open forest, clearing, and forest edge), which is in contrast with previous studies that found that predator activity was mainly concentrated to edge habitats which result in increased predation risk along habitat edges [64,65,66,67,68]. However, the higher predation risk along habitat edges (i.e., edge effect) was mainly recorded within human-dominated landscapes (e.g., farmland [69,70,71], whereas in the forest-dominated landscapes the edge effect is not so evident [72,73]. More specifically, canonical-correlation analysis has suggested that the majority of pass-by-events of mammalian predator were concentrated along forest edges, whereas predation by common raven was recorded in the forest clearings and predation attempts by Eurasian jay in the forest interiors. The predation/foraging activity of individual predator species in individual habitats coincides with previous research that showed that mammalian predator activity is mainly concentrated along habitat edges [65,66,67] whereas common ravens and Eurasian jay search for the prey inside the forest (i.e., within the forest interiors or clearings, see [71,74]). Moreover, we also found a significant effect of the interaction of study area and habitat. Within the forest edge, the difference between study areas was not significant, but we found significant differences between the course of cumulative survival of nests between study areas in the forest interior and clearing. However, it is important to note that these differences were influenced by numbers of predated nests within compared categories. In Jeseníky Mts., we recorded for both habitats only one predated nest. An interesting result is that both these nests were predated at the end of our study, suggesting a lower predation pressure in Jeseníky Mts. compared to Ore Mts.

Our results also suggested that the predation records and predator activity of mammals was generally concentrated to night hours and predation by avian predators was recorded during the day, which match with previous knowledge on avian and mammalian activity patterns. In particular, the activity of mammalian predators (i.e., carnivores and wild boars) is either crepuscular or nocturnal, whereas the avian predators are active during the day hours [75,76].

The composition of predators of artificial nests was different between the study areas. For example, the predation by common raven, Eurasian jay, and wild boar was observed only in Ore Mts., although the occurrence of two latter species was also recorded in Jeseníky Mts. Overall, the stone marten was the most important nest predator in both study areas and was responsible for 39% of depredated artificial nests followed by common raven (25%) and red fox (22%). Red fox was identified as the main nest predator also in previous research [24,37]. Altogether, the red fox and martens were responsible for more than one half of destroyed artificial nests (61%) which is in line with predation on real black grouse nests in Norway [77]. The increasing predation risk on forest grouse populations is more pronounced in recent decades due to increasing population trends of generalist mesocarnivores or corvid species, especially caused by land-use changes or legal protection [8,78,79,80,81].

Wild boar was responsible for 7% of predation cases; however, 18% of predation attempts were conducted by wild boars. Wild boar was also quite frequently detected only passing in front of camera traps (22% of all passes). However, we also recorded large differences in wild boar predation records among study areas. Predation of artificial nests by wild boars was not confirmed by comparable studies previously conducted in the Czech Republic or in the region in south-western Poland bordering with Czech Republic [24,26], despite the fact that the eggs of ground nesting birds are the natural part of wild boar diet [82,83]. Compared to earlier studies, the increase of predation risk could be explained by increasing population trends of wild boar throughout Europe in recent decades [84,85,86,87]. For example, in the Czech Republic, 55,812 individuals of wild boars were hunted in 1990, which increased to 219,277 hunted wild boars in 2017 [45]. The increasing population density and the range expansion of the wild boars into mountain areas could be also partly explained by hunting philosophy [88]. One of common hunting measures is supplementary feeding of ungulates which is common in the context of Central Europe [48] and is practiced in the study area in Ore Mts. The supplementary feeding attracts wild boars into unsuitable mountain areas without sufficient natural food resources [88,89]. Moreover, the nontarget predator species (e.g., red foxes, common ravens) are also attracted into the wider locality with supplementary feeding measures [48,49]. Therefore, the supplementary feeding may increase the predation of a wider group of potential predators to forest grouse nests.

Avian predators were also important nest predators in our study. The average proportion of artificial nests depredated by Eurasian jay and common raven reached 32%. Previous experiments on artificial nest predation in Central Europe show contradictory results. In particular, Svobodová et al. [24] described relatively low predation by avian predators in Ore Mts. compared to results described by Merta et al. [26] in south-western Poland where avian predators and the common raven in particular, depredated 93.9% of artificial nests. High predation caused by common ravens and Eurasian jay could be explained by their dramatically increasing population trends in recent decades [28,45]. In general, avian predators are not considered as important nest predators in Fennoscandia [77]. On the other hand, avian predators (especially common ravens) should be considered among the important predators of black grouse nests in Central Europe [26]. However, it is necessary to mention that the use of artificial nests has obvious shortcomings, e.g., that they are not concealed and protected by the parents like real nests. It is likely to assume that for instance Eurasian jay would have fewer opportunities for predation of black grouse nests guarded by the much larger black grouse female, than for predation of unguarded artificial nests. The predation of artificial nests depredated by smaller avian predators (e.g., Eurasian jay in our case) will probably have a lower impact on the survival of natural nests [35]. In general, the reproductive success measured with the use of artificial nests is frequently underestimated [33]. It can therefore be problematic to assess predation rate of the real nests based on placement of artificial nests. However, the recorded predation of artificial nests is comparable with predation rate of real nests which was mostly studied in Fennoscandia. Similar trends are reported also from Continental Europe and British Isles [8].

## 5. Conclusions

Several studies already confirmed predation as the most important cause of nest failure of forest grouses [16,90,91], which may substantially contribute to their population decline, especially in small and isolated populations. Our study demonstrated the high predation pressure on black grouse nests in the Central European mountain regions which corresponds with increasing population trends of mesopredators and wild boars in Central Europe. Future studies should aim to better understand how the predation risk on black grouse nests is influenced by continuously changing habitat and climate [8] or site-specific long-term changes in predator composition with the increasing populations of generalist predators and potential predation risk by invasive predators [36,50].

## Figures and Tables

**Table 1:** Numbers of predated and non-predated nests within each study area and for three habitat types.

Study Area	Habitat	Predated Nests	Nonpredated Nests	Total
Jeseníky Mts.	Forest edge	7	3	10
	Clearing	1	5	6
	Forest interior	1	5	6
Ore Mts.	Forest edge	4	5	9
	Clearing	8	1	9
	Forest interior	7	3	10
Total		28	22	50

**Table 2:** The effect of study area and habitat type, and its interaction on the course of cumulative survival of nests over time. Cox proportional hazards analysis.

Independent Variable	Wald	df	*p*-Value
**Study area**	5.99	1	0.014
**Habitat** type	0.69	2	0.709
Study area*habitat type	9.00	2	0.011

**Table 3:** The relationships between tested independent variables and distribution of recorded events by artificial nests by each predator species. Canonical correspondence analysis, *n* = 82 recorded events.

Independent Variable	% of Explained Variability	Pseudo-F	*p*-Value
Habitat type	21.7	3.2	0.004
Category of predators’ behavior	18.4	2.8	0.006
Study area	11.3	1.7	0.091

**Table 4:** Numbers of predation events, predation attempts, and pass-by events for each study area and predator species.

Species	Predation			Predation Attempts			Pass-By Events		
	Jeseníky	Ore Mts.	SUM	Jeseníky	Ore Mts.	SUM	Jeseníky	Ore Mts.	SUM
*Martes foina*	8	3	11 (39%)	—	2	2 (10%)	—	5	5 (16%)
*Vulpes vulpes*	1	5	6 (22%)	—	6	6 (27%)	7	8	15 (47%)
*Corvus corax*	—	7	7 (25%)	—	4	4 (18%)	—	—	0
*Garrulus glandarius*	—	2	2 (7%)	5	1	6 (27%)	—	2	2 (6%)
*Sus scrofa*	—	2	2 (7%)	1	3	4 (18%)	2	5	7 (22%)
*Martes martes*	—	—	0	—	—	0	2	—	2 (6%)
*Meles meles*	—	—	0	—	—	0	1	—	1 (3%)

**Table 5:** The effect of day/night and transformed time (interaction between x - hour and y - hour) on presence of bird and mammalian predators at the nests. GLMM analyses, *n* = 107 events. AIC stands for Akaike information criterion.

Independent Variable	df	AIC	% of Explained Variability	Chi	*p*-Value
Null model	3	125.47			
Day/night	4	87.98	33.1	39.49	<0.001
X hour * y hour	6	71.06	50.5	60.41	<0.001

## Data Availability

Full dataset is available at the Forestry and Game Management Research Institute (Czech Rep.).
